# Activation of the Ca^2+^/NFAT Pathway by Assembly of Hepatitis C Virus Core Protein into Nucleocapsid-like Particles

**DOI:** 10.3390/v14040761

**Published:** 2022-04-06

**Authors:** Priya Devi, Tanel Punga, Anders Bergqvist

**Affiliations:** 1 Department of Medical Sciences, Uppsala University, SE 75185 Uppsala, Sweden; priya.devi@medsci.uu.se; 2 Department of Medical Biochemistry and Microbiology, Uppsala University, SE 75123 Uppsala, Sweden; tanel.punga@imbim.uu.se; 3 Clinical Microbiology and Hospital Infection Control, Uppsala University Hospital, SE 75185 Uppsala, Sweden

**Keywords:** HCV, core protein, SPP cleavage, ER, calcium, NFAT, virus assembly, nucleocapsid-like particle

## Abstract

Hepatitis C virus (HCV) is the primary pathogen responsible for liver cirrhosis and hepatocellular carcinoma. The main virion component, the core (C) protein, has been linked to several aspects of HCV pathology, including oncogenesis, immune evasion and stress responses. We and others have previously shown that C expression in various cell lines activates Ca^2+^ signaling and alters Ca^2+^ homeostasis. In this study, we identified two distinct C protein regions that are required for the activation of Ca^2+^/NFAT signaling. In the basic N-terminal domain, which has been implicated in self-association of C, amino acids 1–68 were critical for NFAT activation. Sedimentation analysis of four mutants in this domain revealed that association of the C protein into nucleocapsid-like particles correlated with NFAT-activated transcription. The internal, lipid droplet-targeting domain was not required for NFAT-activated transcription. Finally, the C-terminal ER-targeting domain was required in extenso for the C protein to function. Our results indicate that targeting of HCV C to the ER is necessary but not sufficient for inducing Ca^2+^/NFAT signaling. Taken together, our data are consistent with a model whereby proteolytic intermediates of C with an intact transmembrane ER-anchor assemble into pore-like structures in the ER membrane.

## 1. Introduction

**Figure 1:**
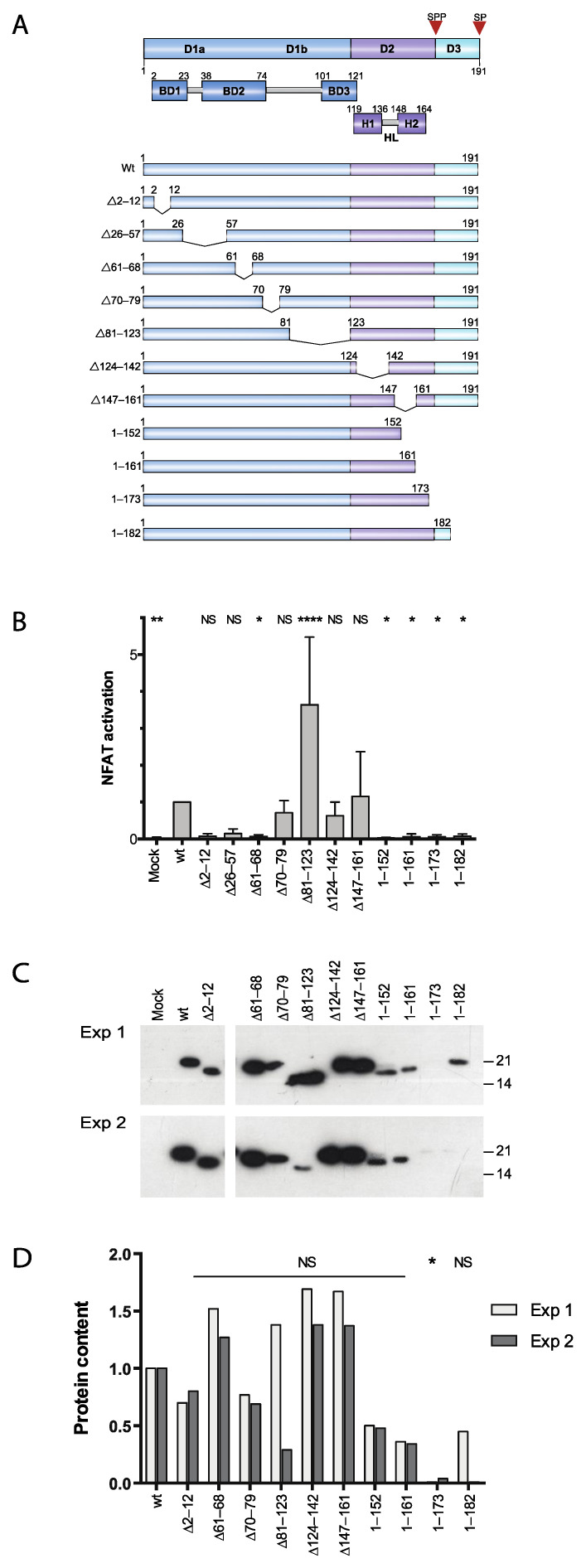
Activation of NFAT by HCV C deletion mutants. (**A**) Schematic representation of the HCV Core (**C**) protein domains and deletion mutants. The C protein is divided into 3 domains, D1, D2 and D3. Based on the distribution of charged amino acid, D1 is further subdivided into three basic domains, BD1, BD2 and BD3. D2 comprises two amphipathic alpha-helices helix 1(H1) and helix 2(H2) connected by a hydrophobic loop (HL). D3 is a transmembrane domain (TM) that contains signal sequences and is processed by host proteases. The first cleavage of the C protein at amino acid (aa) residue 191 by the host signal peptidase (SP) releases immature C protein (p23) of 191 aa. The second cleavage by ER resident signal peptide peptidase (SPP) releases the mature C protein (p21) of 177 aa. Different deletion mutants are generated by deleting the defined aa sequences in D1, D2 and D3 within the C protein as shown in the figure. Deletions are represented by Δ and the number corresponds to the aa removed in the C protein deletion mutants. (**B**) Transcriptional activation by the C protein deletion mutants. Jurkat cells were co-transfected with an NFAT-luc reporter plasmid together with a plasmid encoding either full-length C (wt) or various mutants. As a negative control, an empty vector was used (Mock). At 40 h post-transfection, cells were stimulated with TPA for 7 h and the relative luciferase activity was determined. Values are normalized to the positive control. Data are presented as mean ± standard deviation (*n* = 3–7). (**C**) Expression of the C mutant proteins in Jurkat cells. Transiently transfected Jurkat cells were analyzed with respect to the C protein content by Western blotting with anti-HCV C antibody. The positions of molecular weight markers (kDa) are indicated to the right. Uniform gel loading was verified by staining the membrane for total protein content with Ponceau S prior to immune detection. Expression of deletion mutant ∆26–57 was not verified since it is not recognized by the anti-HCV C antibody. (**D**) Relative protein content from two independent runs were determined by densitometry. The statistical significance was determined by a one-way ANOVA test with Dunbar’s correction and bars with stars were statistically significant with adjusted *p* values; * *p* < 0.05, **** *p* < 0.0001.

Hepatitis C virus (HCV) is the major cause of nonparenteral non-A non-B hepatitis [1,2]. Characteristic features of HCV infection include a high incidence of persistent infection resulting in chronic hepatitis, which is a strong risk factor for liver cirrhosis and hepatocellular carcinoma. The propensity of HCV to persist in the vast majority of infected individuals suggests that the virus has evolved mechanisms of evading host immunity. HCV is an enveloped, single-stranded positive-sense RNA-virus belonging to the Flaviviridae family [3,4]. The HCV genome comprises approximately 9600 nucleotides and is translated into a polyprotein of about 3000 amino acids (aa). The polyprotein is cleaved by cellular and viral proteinases into three structural proteins and seven non-structural proteins. Among the HCV gene products, the core protein (C) has been implicated in both oncogenesis and immune subversion. The C protein is cleaved from the N-terminus of the 3011 aa long polyprotein precursor by host signal peptidase located in the endoplasmic reticulum (ER), resulting in a 191 aa polypeptide, p23. Subsequently, p23 remains anchored to the ER through a C-terminal region and is further processed at its C-terminus by signal peptide peptidase, leading to a mature protein composed of the N-terminal ~177 aa residues, referred to as p21 [5,6]. Based on the hydrophobicity pattern and the clustering of basic amino acids it is possible to separate the C protein into three domains (Figure 1A). Domain 1 consists of residues 1 to 117 and contains a high proportion of basic residues along with two short hydrophobic domains. Based on similarity with Flaviviruses, domain 1 can be further divided into a conserved subdomain 1a and subdomain 1b, which is unique for HCV. Domain 2 (aa 118–177) has a significantly lower proportion of basic residues and is more hydrophobic than domain 1. Domain 3 (aa 178–191) is highly hydrophobic and acts as the signal sequence for the HCV protein E1 [7]. HCV C assembly and formation of nucleocapsid structure include interaction with viral RNA, oligomerization via specific C-C interactions and interaction with the E1 protein. In addition to interactions required for virus assembly, the C protein has been reported to interact with a number of cellular proteins, including RNA-binding proteins and members of the TNF receptor family (reviewed in [8,9]. Although the primary target for HCV is the hepatocytes, there is increasing evidence that the virus is capable of infecting other sites, including the heart [10], lymph nodes [11,12] and PBMCs in vivo and in vitro [13,14,15,16,17].

HCV is an oncogenic virus associated with liver steatosis and hepatocellular carcinoma [18]. Based on animal models, expression of the C protein is sufficient to induce steatosis and liver cancer [19,20]. Biochemically, the C protein is associated with several stress responses [21,22,23,24], including ER stress [25,26], oxidative stress [27,28,29] and unfolded protein responses [25,30]. We and others have previously shown that expression of the C protein in different cell lines triggers calcium (Ca^2+^) signaling and alters Ca^2+^ homeostasis [26,31,32]. In immortalized T lymphocyte (Jurkat) cells, expression of the C protein promotes Ca^2+^ leakage from the intracellular stores and causes Ca^2+^ oscillations that favor the activation of transcription factor NFAT-regulated promoters, including IL-2 [32,33]. Interestingly, the effects of C on Ca^2+^ signaling are resistant to inhibition of upstream signaling, suggesting that C may act directly on integrity of the ER membrane. 

In this report, we performed a mutational analysis of the C protein to correlate its effects on Ca^2+^/NFAT-signaling with intracellular localization and assembly into nucleocapsid-like particles (NLPs). We identified two C protein separate elements that are critical for NFAT-activated transcription. Our results indicate that targeting of HCV C to the ER is required but not sufficient for inducing Ca^2+^/NFAT-signaling, and is consistent with a model where the p23 intermediate with an intact transmembrane ER-anchor assembles into pore-like structures in the ER membrane. 

## 2. Materials and Methods

### 2.1. Chemicals

All chemicals used were of analytical grade. Restriction endonucleases, T4 DNA polymerase, Taq polymerase and T4 DNA ligase were obtained from MBI Fermentas. Chemicals 12-O-tetradecanoylphorbol 13-acetate (TPA) and MG132 were from Sigma-Aldrich (Stockholm, Sweden). TurboFectTM and Lipofectamine™ transfection reagents were purchased from Thermo Fisher Scientific (Uppsala, Sweden). Horseradish peroxidase-linked anti-immunoglobulins were from Dako A/S (Sundbyberg, Sweden). The monoclonal antibody C7-50 [34] recognizing HCV C was from Santa Cruz Biotechnology (Heidelberg, Germany). 

### 2.2. Cells, Cell Culture and Viral Genomes

The human Jurkat T cell (subclone E6-1) was obtained from the American Tissue Type Collection. HeLa T-REx cells were provided by Stephen Taylor [35]. Clontech’s HeLa Tet-On™ cells were purchased from. Takara Bio Europe SAS (Saint-Germain-en-Laye, France). Lymphoid Jurkat cells were cultured in RPMI-1640 medium whereas HeLa cells were propagated in DMEM medium. All media were from Sigma-Aldrich and supplemented with 10% fetal bovine serum from Fisher Scientific (Gothenburg, Sweden) and 1 % penicillin-streptomycin mixture (Sigma-Aldrich).

Plasmids encoding mutated core protein with internal deletions were made either by removal of restriction fragments from the expression plasmid pOP/HCV1-194 [33], or by generating DNA fragments with internal deletions using polymerase chain reaction (PCR). The plasmid pHCVC∆81–123 was made by deletion of the Kpn I-Cla I fragment followed by flush-end ligation. The plasmid pHCVC∆124–142 was made by deletion of the Cla I-Xag I fragment. To retain the correct reading frame a Not I linker (AGCGGCCGCT) was ligated to the flushed ends prior to recircularization, resulting in a conservative substitution of D124 to E124 and an insertion of an arginine. The constructs pHCVC∆2–25, pHCVC∆2–12, pHCVC∆26–57, pHCVC∆61–68, pHCVC∆70–79, pHCVC∆147–161, pHCVC1–161pHCVC1–173 and pHCVC1–182 were made by PCR amplification. Purified fragments were cleaved with Eco RI, Avr II, Nde I, Kpn I, Cla I or Xag I and cloned into the corresponding sites of pOP/HCVC1–194 [33] or pCN/HCVC1–194 (A. Bergqvist and C.M. Rice, unpublished) (Table 1). The plasmids encoding signal sequence mutants spmt1–191 and spmt1–194 were made by overlap extension PCR using pSV/spmt as template [5]. The expression plasmid pOP/HCV1–152 [33] and the luciferase reporter plasmid NFAT-luc [36] have been described earlier. NFAT-luc contains three copies of the NFAT site (−286 to −257 of the human IL-2 gene) linked to the human IL-2 promoter (−72 to +47) driving firefly luciferase expression.

### 2.3. Analysis of Gene Expression

Jurkat cells and derivatives thereof were transfected using the DEAE-dextran procedure as previously described [33]. An equal amount of effector and luciferase reporter plasmid DNA was used. The cells were aliquoted at 40 h post transfection and stimulated with 50 ng/mL TPA. Seven hours later the cells were washed once in phosphate-buffered saline (PBS) and lysed in a buffer containing 25 mM Tris-phosphate (pH 7.8), 2 mM 1,4-Dithiothreitol (DTT), 10% glycerol and 1% Triton X-100. Luciferase and activity were measured with a Sirius luminometer from Berthold Detection Systems using beetle luciferin from Promega (Stockholm, Sweden). Statistical significances were determined by one-way ANOVA test with Dunbar’s correction.

### 2.4. Preparation of Cell Lysate, SDS-PAGE and Western Blotting

The protein expression of HCV C mutants was analyzed by transient transfections. HeLa T-REx (2 × 10^5^) cells were transfected with 2 µg DNA in the presence of 8 µL TurboFect^TM^ transfection reagent (Thermo Fisher Scientific). Forty-eight hours post-transfection, the growth medium was removed and the cells were washed with PBS. Cells were scraped into PBS and the detached cells were centrifuged at 200× *g* for 10 min at 4 °C. The cell pellets were suspended in SDS-PAGE sample buffer or lysed in NP-40 buffer (50 mM Tris-Cl, pH 7.5; 150 mM NaCl and 1% NP-40), incubated on ice for 20 min and clarified by centrifugation at 13,000 rpm for 15 min at 4 °C. The protein concentration was determined by Bio-Rad protein assay (Solna, Sweden). Equal concentrations of protein samples were resolved on homemade 12% SDS-PAGE gel and transferred onto a 0.2 µM Amersham nitrocellulose membrane (GE Healthcare, Uppsala, Sweden) using the wet tank transfer approach. The membranes were blocked with Odyssey blocking buffer (LI-COR Biotechnology, Homburg Germany) at RT for 1 h prior to overnight incubation with primary antibodies against HCV C (anti-Hep C cAg C7-50 (1:200; Santa Cruz, Sc-57800)) and actin (1:1000; LI-COR) at 4 °C. The membranes were washed with 0.2% Tween 20 in PBS (TPBS) and incubated with secondary horseradish peroxidase-linked (Dako A/S) or by fluorescent labeled secondary antibodies (IRDye^®^, LI-COR 1:5000) for 1 h in the dark. Washed membranes were visualized with Pierce enhanced chemiluminescence (Thermo Fisher Scientific) or scanned using an odyssey CLX imaging system (LI-COR). Relative protein contents were determined with optical densitometry by using the software Image J version 1.53.

### 2.5. Treatment with MG132

When treated with MG132 (Sigma-Aldrich), cells were incubated for 44 h after transfection at 37 °C and supplemented with fresh growth medium containing MG132 at the final concentration of 10 µM. Incubation was continued at 37 °C for an additional 4 or 16 h before the cells were harvested and prepared for SDS-PAGE and Western blot analysis.

### 2.6. Indirect Immunofluorescence 

HeLa T-REx (1 × 10^5^) cells grown on 13 mm coverslip were transfected for 48 h with indicated plasmids using TurboFect^TM^ transfection reagent (Thermo Fisher Scientific) according to the manufacturer’s instructions. Two days after transfection, cells were washed twice with PBS and fixed in ice-cold acetone/methanol (50/50) for 20 min. After washing with PBS, cells were incubated with blocking solution (5% fetal bovine serum (FBS) and 0.3% Triton X-100 in PBS) for 1 h. Thereafter, the coverslips were incubated on a piece of parafilm with 50 µL primary antibodies solution diluted in PBS-1% FBS in a humidified chamber for 1 h at room temperature. The following primary antibodies were used: anti-Hep C cAg C7-50 (1:200; Santa Cruz, Sc-57800), anti-ADFP (1:300; ab78920) from Abcam (Amsterdam, Netherlands) and anti-calnexin (1:500; PA5-34754) from Invitrogen (Uppsala, Sweden). After washing with PBS, cells were incubated with secondary antibody diluted in PBS-1% FBS in the dark for 1 h. The fluorescent secondary antibodies were Alexa Fluor 568- or Alexa Fluor 488-conjugated anti-mouse or anti-rabbit IgG (H & L) antibodies (1:1000; Abcam, ab175473 and ab150081, respectively). After extensive washing with PBS, coverslips were placed on glass slides with mounting media supplemented with DAPI (Abcam) for nuclear staining. Immunolabeled cells were visualized under a fluorescence microscope from Olympus (LRI Instrument AB, Lund, Sweden) and images were captured with INFINITY ANALYZE and CAPTURE (Lumenera Corporation, version 6.5, Ottawa, Canada) and analyzed with Adobe Photoshop, version 10.0.1. 

### 2.7. Centrifugation Analysis of HCV Nucleocapsid-like Particels

HeLa Tet-On cells were transfected with indicated plasmids using Lipofectamine transfection reagent according to the manufacturer´s instruction. After 40 h, cells were lysed in a buffer containing 10 mM Tris-Cl, pH 7.5; 50 mM NaCl; 10 mM KCl, 5 mM MgCl_2_, 2 mM DTT and 1% NP-40. For sedimentation analysis, the clarified cellular extract was layered onto a 5–20% sucrose gradient made of lysis buffer and containing a 55% sucrose cushion at the bottom. After ultracentrifugation in a Sorvall TH-660 rotor at 45,000 rpm at 4 °C for 30 min, nine 500 µL fractions were collected from the bottom. For determination of buoyant densities, the cellular extracts were extracted with chloroform to remove lipids and subjected to ultracentrifugation in a SW55 rotor at 36,000 rpm at 25 °C for 24 h in the presence of CsCl (0.4 g/mL). Eight fractions were collected from the bottom. In both cases, the protein content was precipitated with 8% trichloroacetic acid and analyzed by Western blotting.

### 2.8. Electron Microscopy

Cells were subjected to velocity sedimentation centrifugation, and three intermediate fractions were pooled and dialyzed for 18 h against phosphate-buffered saline at 4 °C. Dialyzed samples were then settled onto carbon-coated grids for 2 to 3 min, stained with 1% uranyl acetate for 30 s, and visualized with a Zeiss EM 10C/CR transmission electron microscope (Stockholm, Sweden). Studies were performed using a Philips CM100 electron microscope (Philips, Eindhoven, The Netherlands). After fixation in 1.25% glutaraldehyde, virus particles were pelleted on carbon/Formvar-coated 400-mesh copper grids (GilderGrids, Lincolnshire, UK). Briefly, 150 μL of virus suspension was centrifuged for 10 min in an Eppendorf 5417C centrifuge (Hamburg, Germany) with a swing-out rotor at a maximum force of 12,000× *g*. The grids were placed on the flat bottom of the outer container of Sarstedt microvette CB 300 tubes (Nümbrecht-Rommelsdorf, Germany). Grids were stained by 2% tungstophosphoric acid (Merck, Darmstadt, Germany) at pH 6.

## 3. Results

### 3.1. Two Distinct Regions of C Are Required for Activation of Ca^2+^/NFAT-Signaling

We previously showed that expression of the HCV C protein promotes the leakage of Ca^2+^ from intracellular stores and favors the activation of NFAT-regulated promoters [32]. To investigate the mechanism of the C protein-induced Ca^2+^/NFAT signaling by correlating this activity with intracellular localization and other biochemical properties, a set of deletion mutants encompassing the entire C was generated (Figure 1A). Jurkat cells were co-transfected with an effector plasmid expressing full-length or mutated C protein together with a luciferase reporter plasmid driven by three copies of the distal composite NFAT/AP1-element from the IL-2 promoter. At 40 h post transfection, cells were stimulated with TPA, and 7 h later the cells were lysed and the luciferase activity was determined. Using different C protein deletions, we found that whereas the central part of the polypeptide was dispensable, the N-terminal half and the C-terminal half of C were required for activation of NFAT-driven transcription (Figure 1B). Deletions in the N-terminal region of C were detrimental for activation of NFAT-driven transcription (∆2–12, ∆26–57 and ∆61–68). The internal deletions in the region aa 70–161 were either not significantly different from full-length C (∆70–79, ∆124–142 and ∆147–161) or displayed a three-fold increase in reporter gene activation (∆81–123). Finally, all the C-terminally truncated forms of the C protein (1–152, 1–161, 1–173 and 1–182) were unable to activate NFAT-driven transcription. 

To examine whether expression levels or protein stability could account for the different effects observed on reporter gene activation, the C protein levels were analyzed in transfected Jurkat cells by Western blotting and determination of relative protein content by densitometry. Based on these experiments, the expression levels of C with internal deletions in domain 1 and 2 were not significantly different from those of full-length protein (Figure 1C,D). Whereas two of the C-terminal truncations, 1–152, 1–161, were lower albeit clearly detectable, the distal deletions 1–173 and 1–182 were barely visible and just above the detection limit (Figure 1C,D). Of these, only the decrease of 1–173 was considered significant.

### 3.2. Effect of Proteasome Inhibiton on Expression of HCV C Proteins

**Figure 2:**
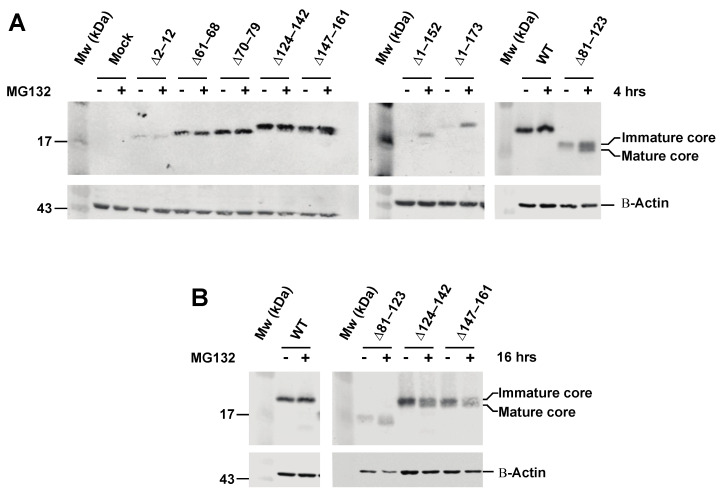
Expression and stability of HCV C mutant proteins. (**A**) HeLa T-REx cells transfected with plasmid encoding either full length (wt) or mutant C proteins. After 48 h, cells were lysed and presence of C was detected by Western blotting. In panel A, (+) indicates lanes; cells were treated with 10 µM MG 132 for 4 h before harvesting. (**B**) HeLa T-REx cells transfected with plasmid encoding C mutants ∆81–123, ∆124–142 and ∆147–161 were treated with 10 µM MG132 for 16 h. Actin was used as loading control. The positions of molecular weight markers (kDa) are indicated to the right.

To further examine expression of the C proteins, highly transfectable HeLa T-Rex cells were transiently transfected with different C expressing plasmids followed by Western blotting. Most of the C proteins were well-expressed, with the exception of the ∆2–12, 1–152 and 1–173 mutants, which showed very low expression (Figure 2A). Since low protein detection may indicate that the C proteins are degraded by the proteasome, we treated cells with MG132, a known proteasome inhibitor. Immunoblot analysis showed that MG132 treatment did not increase accumulation of the C protein with deletions in the N-terminal part of domain 1 (∆2–12, ∆61–68 and ∆70–79). Notably, the mature form of the domain 1 mutant ∆81–123 accumulated considerably more in MG132-treated than untreated cells (Figure 2A,B). The mutant 1–152 was detected in MG132-treated but not in untreated cells, whereas the C mutant 1–173 showed low detection in untreated cells and its accumulation increased moderately due to MG132 treatment. Whereas the effect of MG132 of treatment of domain 2 mutants (∆124–142 and ∆147–161) was limited after 4 h (Figure 2A), a significant increase in the mature form of C accumulated after 16 h (Figure 2B). Together, these results suggest that the sequence from aa 81–123 and downstream is essential for protein stability and that the C-terminal part of domain 1 (aa 81–123, and domain 2 (aa 124–142 and aa 147–161) is required for maturation (Figure 2A,B).

### 3.3. Activation of Ca^2+^/NFAT Signaling Is Unrelated to the C Protein Subcellular Localization

**Figure 3:**
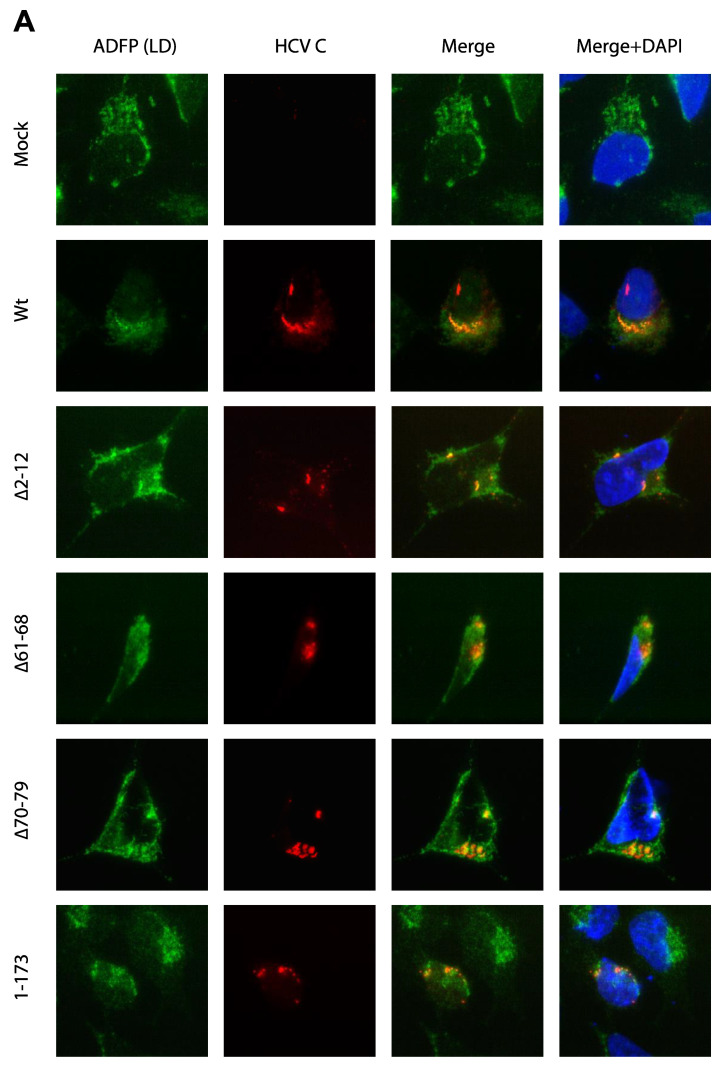

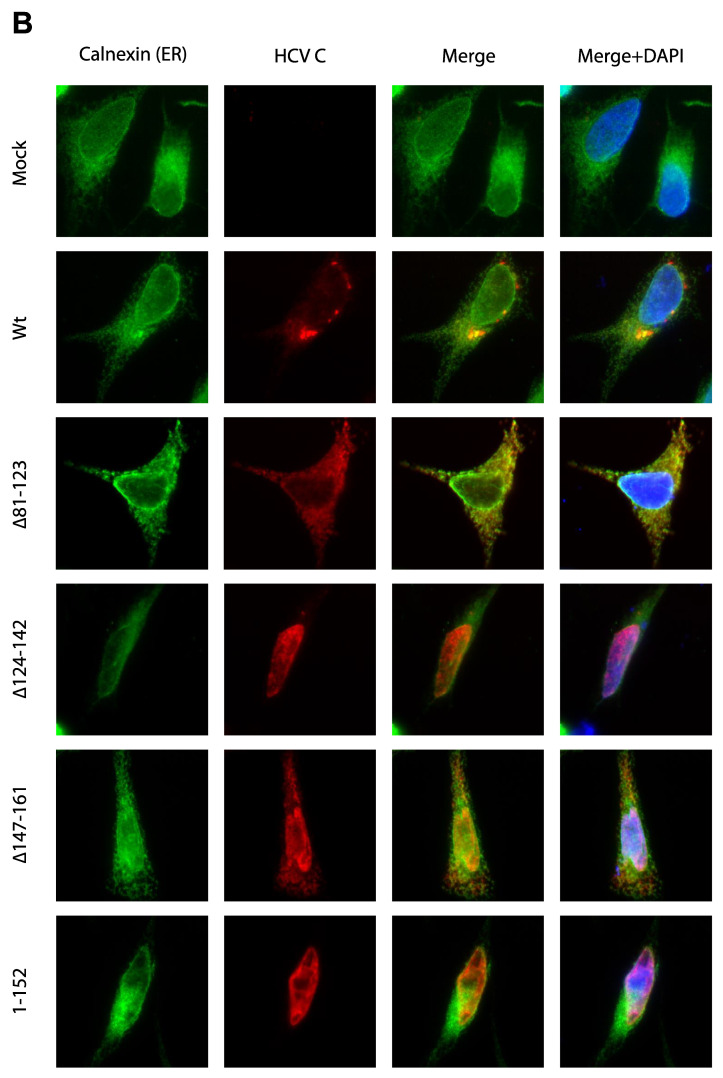
Intracellular localization of the C mutant proteins. HeLa T-REx cells transfected with plasmid encoding either the wild-type (wt) or selected C mutant proteins. (**A**) At 48 h post-transfection, cells were fixed in acetone/methanol and labeled with the anti-HCV C and anti-ADFP (a cellular marker for lipid droplet), and were analyzed by indirect immunofluorescence assay. Nuclei were visualized after staining with DAPI. (**B**) The C protein distribution in ER or nucleus was detected in the C deletion mutant protein ∆81–123, ∆124–142, ∆147–161 and 1–152 expressing cells. Cells were stained with the anti-HCV C (red) and anti-calnexin (ER marker) green antibodies. Nuclei were visualized after staining with DAPI.

Activation of NFAT is likely a consequence of the fact that expression of C is associated with increased cytoplasmic Ca^2+^, which in turn leads to the activation of calcineurin and dephosphorylation and nuclear translocation of NFAT. Previous reports have shown that the C protein is mainly targeted to lipid droplets, a globular storage compartment that stores triacylglycerol and cholesterol esters, and also to ER, which serves as the main store of intracellular Ca^2+^ [6]. To correlate the distribution of C mutants with NFAT activation, colocalization of C with ADFP (a cellular marker for lipid droplet) or calnexin (an ER-resident protein) was analyzed by indirect immunofluorescence. The full-length C (wt) co-localized with ADFP and demonstrated a globular ring-like staining pattern in the perinuclear region corresponding to lipid droplet at higher magnification in HeLa T-Rex cells. HCV C deletion mutants (∆2–12, ∆61–68, ∆70–79) and the C-terminal truncation mutant (1–173) displayed co-localization with ADFP similar to that of the full-length C protein. A ring-like staining pattern was also observed in the mutants ∆61–68 and ∆70–79 expressing cells (Figure 3A). Due to very intense staining of C in the lipid droplet compartment, the exposure had to be adjusted accordingly and cannot thus be directly compared with the images from other compartments. Deletion mutant ∆81–123 displayed the punctate cytoplasmic staining and co-localized with ER marker protein calnexin (Figure 3B). Deletions of the amino acid residue in domain 2, ∆124–142 mutant and ∆147–161 were found predominantly in the nucleus but also in cytoplasm co-localized with calnexin. The C-terminal deletion 1–152 lacking the entire ER anchor sequence was found in the nucleus.

### 3.4. The Intermediate p23 Form of HCV C Is Responsible for Activation of Ca^2+^/NFAT-Signaling

**Figure 4:**
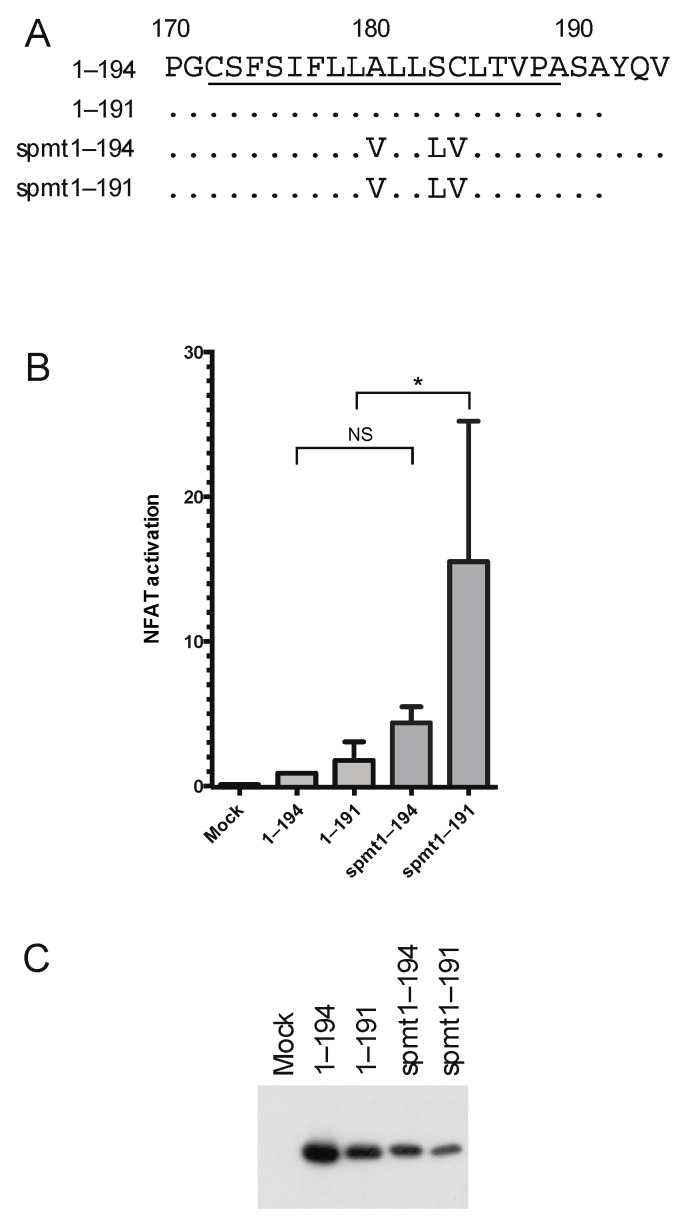
Analysis of the transmembrane region of HCV C for activation of Ca^2+^/NFAT. (**A**) A schematic map of the C-terminal part of HCV C D3. Amino acids in the transmembrane region are underlined. The positions of amino acid substitution in the spmt mutants are indicated. (**B**) Activity of the HCV C transmembrane mutants. Relative transcriptional activity of the spmt mutants was determined using NFAT-luc reporter assay as shown in Figure 1B. The sample expressing the 1–194 protein was taken as 1. Data are presented as mean ± standard deviation (*n* = 3). The statistical significance was determined by one-way ANOVA test with Dunbar’s correction and bars with stars were statistically significant with adjusted *p* values; * *p* < 0.05. (**C**) Expression of the spmt mutants was verified by Western blotting using anti-HCV C antibody. Uniform loading of the gel was verified by staining the membrane for total protein content with Ponceau S prior immune detection.

The results from the HCV C deletion mutants suggest that the entire C-terminal transmembrane region, domain 3, is required for activation of Ca^2+^/NFAT-signaling and that the effect is mediated by the intermediate p23 form rather than the mature p21 protein. To further analyze the activity of the p23 protein, we took advantage of the C mutant spmt, which due to the 3 aa substitutions in the transmembrane region is not processed by the peptidase (Figure 4A) [5]. To exclude that the effects on Ca^2+^/NFAT-signaling were due to the presence of additional aa downstream of the signal peptidase cleavage site at 191, constructs with authentic ends were made and compared with the full-length C (1–194). The mutants in the transmembrane region, spmt 1–194 and spmt1–191, which are not processed into the mature p21 form, activated 5- and 10-fold more of the NFAT-driven reporter gene than their counterparts with the wild-type transmembrane region (Figure 4B). Although the activation by C with authentic ends C1–191 was slightly higher, this effect was not significant. The expression levels and integrities of spmt mutants in comparison with wild type were verified by Western blotting (Figure 4C).

### 3.5. Association of the C Protein into Nucleocapsid-like Particles Correlates with Activation of the NFAT Signaling

**Figure 5:**
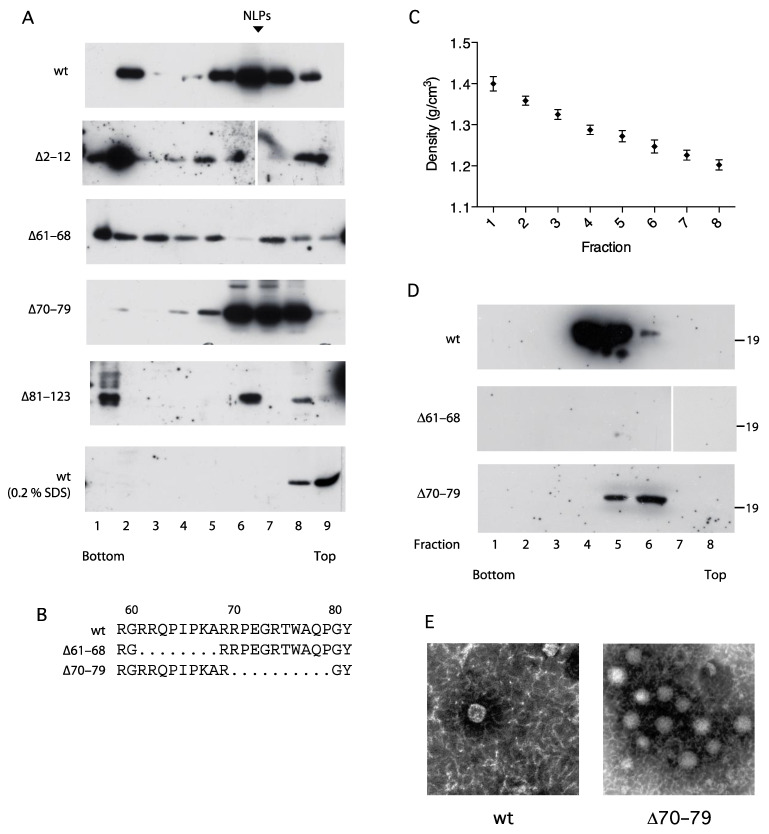
Analysis of nucleocapsid-like particles. HeLa Tet-On cells were transfected with plasmids expressing either full-length (wt) or C deletion mutants (∆61–68 or ∆70–79). (**A**) Sedimentation analysis of wt (1–194) and C mutants. Cellular extracts were loaded on 5–20% sucrose gradients and subjected to ultra-centrifugation. The gradients were fractionated and analyzed for the C protein accumulation as described above. The expected positions of nucleocapsid-like particles (NLPs) are indicated by arrows. (**B**) Map of internal part of D1. Amino acid deletions in ∆61–68 and ∆70–79 are indicated. (**C**) The densities of C containing fractions were determined by refractometry. (**D**) Buoyant density analysis of wt (1–194) and C mutants. Cellular extracts were mixed with CsCl and subjected to ultracentrifugation. The gradients were fractionated and the C protein was then detected by Western blotting using an anti HCV C antibody. The order of the samples is indicated with numbers from the bottom to the top of the gradient. (**E**) Electron microscopy of the NLPs. Fractions positive for NLPs in sedimentation centrifugation were dialyzed, negatively stained and examined by transmission electron microscopy.

The C protein regions within domain 1a (aa 36–91, aa 82–102 and aa 1–75) have previously been reported to be essential for self-association into nucleocapsid-like particles (NLPs) [37,38,39,40]. To further investigate the mechanism for C-induced activation of NFAT, four C mutants with deletions in domain 1a and 1b were characterized with respect to their capacity to self-associate into nucleocapsid-like particles (Figure 5A). Extracts from transfected HeLa cells were subjected to isopycnic and zonal-rate ultracentrifugation and collected fractions were analyzed with respect to C content by Western blotting. Sedimentation analysis in sucrose gradients revealed that, under native conditions, full-length C was predominantly found in the intermediate fractions (5–8) at the expected position of NLPs (Figure 5A). A minor fraction conceivably reflecting large aggregates was found on top of the heavy sucrose cushion in fraction 2. In the presence of 0.2% SDS, HCV C was localized in the top fractions indicating disruption of NLPs into monomers. Whereas ∆70–79 was indistinguishable from the full-length C and ∆81–123 was predominantly found in fraction 6, ∆2–12 and ∆61–68 were distributed across the entire gradient with no accumulation in the intermediate fractions. 

For further analysis of their biochemical properties, two of the C mutants, ∆61–68 and ∆70–79, were selected. These mutants have adjacent deletions in domain 1a and displayed similar expressions levels (Figure 1D) and stability (Figure 2A), and identical intracellular localization (Figure 3A), but have different effects on NFAT activation (Figure 1B) and sedimentation rate (Figure 5A). Isopycnic centrifugation in CsCl gradients revealed accumulation of full-length C at a density of 1.28 g/mL (Figure 5C). Whereas ∆70–79 was slightly lighter than wild type, no accumulation of ∆61–68 in the intermediate fractions was observed. 

To confirm the biochemical data suggesting that the HCV core forms NLPs, pooled fractions containing C from the velocity sedimentation were analyzed by transmission electron microscopy. NLPs were identified with wt and ∆70–79 (Figure 5E). The size and morphological appearance were consistent with what others have reported for HCV capsids isolated from insect cells, chimpanzees and infected humans [40,41,42]. 

## 4. Discussion

We previously showed that expression of HCV C in Jurkat cells alters T-cell receptor-triggered Ca^2+^ oscillations that, during transient expression, favor selective activation of NFAT-dependent gene expression. Although truncated forms of HCV C lacking the C-terminal domain have been shown by others to activate other cellular and heterologous viral promoters, the C-terminal region of the C protein is required for activation of the IL-2 promoter [33]. Since the effects of the C protein on Ca^2+^ signaling were resistant to inhibition of the inositol-3-phosphate gated channel and upstream signaling and the C-terminal end is required, we hypothesized that C may act directly on the integrity of the ER membrane. To further investigate the mechanism of the C-induced Ca^2+^ signaling, a set of C deletion mutants was generated and characterized with respect to intracellular localization. As a read-out for Ca^2+^ signaling, we measured expression of the transcription factor NFAT-controlled reporter gene (Figure 1B). We found that whereas deletions in the N terminal part of domain 1 (∆2–12, ∆61–68) and the truncations of ER-anchoring domain (1–152, 1–161, 1–173 and 1–182) were detrimental for activation of Ca^2+^ signaling, deletions in the distal part of domain 1 (∆70–79, ∆81–123) and domain 2 (∆124–142, ∆147–161) indicate that these regions are not essential. To ensure that the effects observed on activation of NFAT were not due to differences in protein expression, the protein content for each mutant was quantified by optical densitometry of two independent runs. Although this procedure has a limited dynamic range, it was nevertheless sufficient to conclude that the observed effects on NFAT of the internal mutants ∆2–12, ∆61–68 and ∆81–123 could not be explained by alterations in protein levels (Figure 1C,D). For the C-terminal mutants, defective NFAT-activation was associated with reduced steady-state levels of protein. Although the C-terminal mutants 1–152 and 1–161 were clearly detectable, we cannot exclude the possibility that lower expression of these mutants contributed to the abolished activity (Figure 1C,D). 

Next, we analyzed the intracellular location of the C mutants (Figure 3A). This has been extensively investigated by others with widefield and confocal microscopy, and our results are essentially in line with previous observations [5,43,44,45,46]. The mutants with deletions in the N terminal part of domain 1 (∆2–12, ∆61–68, ∆70–79) and the ultimate C-terminus (1–173) displayed dense accumulation in lipid droplets similar to the full-length C protein (Figure 3A). However, several outliers from this pattern were observed. First, the C mutant with deletion of the internal aa 81–123 was found in a reticular pattern over the entire cytoplasm corresponding to the ER compartment (Figure 3B), which is line with previous observation that deletion of aa 124–142 abrogates targeting to lipid droplets [6]. Past studies have shown that residues between 153 and 169 are critical for lipid droplet association, whereas another study revealed that the residues between 119–136, 124–142 and 148–164 corresponding to domain 2 of C protein form the alpha helix and are linked by a hydrophobic loop [5,6,45]. Our results from mutant ∆124–142 and ∆147–161 is also in line with previous findings and indicates that the truncations in this region disrupt the ability of C to associate with lipid droplets [6]. Nuclear localization of these mutants may be a result of permeabilization of nuclear membrane by methanol used in fixation, as also observed in other study [6] Alternatively, nuclear localization may be a result of overloading of the intracellular trafficking system due to overexpression or by unmasking of a nuclear localization in domain 1, which is normally overcome by targeting signals in domains 2 and 3 [47,48,49]. Finally, truncation of the entire ER-anchoring C terminal domain 3 was associated with nuclear localization (Figure 3B). 

When the C mutants were characterized with respect to activation of Ca^2+^/NFAT signaling, the N terminal amino acids 1–68 and an intact domain 3 were found to be essential. Whereas one of the inactive mutants in the C-terminus, 1–152, was localized in the nucleus, 1–173 and the mutants in the N-terminal region were localized in the same compartment as full-length C, indicating that altered localization in some but not all cases correlated with compromised activity. Moreover, the mutants found in the ER but not in lipid droplets were all activating NFAT; in fact, ∆81–123 was even more potent than full-length C. Notably, this mutant was poorly processed in the C-terminus and had an intact transmembrane region (Figure 2). Thus, whereas targeting of the C to the nucleus or lipid droplets did not correlate with activation of NFAT, localization of HCV C to the ER seemed to be necessary although not sufficient for activation of NFAT-mediated transcription. The observation that the entire ER-targeting signal, which is partially removed by proteolytic processing to produce mature C, was required *in extenso* suggests that the effect on NFAT was attributed to the p23 intermediate rather than mature C. This idea is further supported by the observations that increased activity was obtained by the ∆81–123 and with the spmt mutants (Figure 4), which do not undergo signal peptide processing and thus retain the entire transmembrane region.

Sedimentation analysis of four mutants with deletions in domain 1a and 1b revealed that aa 1–12 and 61–68 but not aa 70–79 and 81–123 were critical for assembly into NLPs, which is in line with previous findings that identified the aa critical for C-C interactions within domain 1a [37,38]. Since ∆2–12 and ∆61–68 were also defective in activation of NFAT-mediated transcription, a correlation with this activity and the ability of C to associate into NLPs were observed. Notably, although aa 1–124 is sufficient for association of NLPs in vitro, the partially processed p23 intermediate is required for association into NLPs in cell culture [50]. A conceivable hypothesis is thus that subsequent budding of assembled NLPs through intracellular membranes is accompanied by disturbed membrane integrity, ultimately causing increased permeability to Ca^2+^. Alternatively, association of several C molecules with intact trans-membrane C-terminus may result in formation of pore-like structures. Multimers of specific virus proteins, so-called viroporins, comprising two amphipathic/transmembrane alpha helices, can form pore-like structures on the ER membranes [51]. Hence, it remains to be tested whether the C protein indeed can function as a classical viroporin. 

Several of the HCV proteins, including C, E1, E2, p7, NS2, NS4B and NS5B, comprise transmembrane regions [52]. Of these, p7 has previously been demonstrated to form ion channels and act as a viroporin [51,53,54,55,56]. Of the remaining HCV proteins, C forms highly ordered, oligomeric structures that would facilitate formation of such complexes [37]. In addition to Ca^2+^ signaling, expression of the C protein is also associated with ER stress and oxidative stress, and unfolded protein responses. Since all these phenomena are tightly linked to the ER membrane, it is not unlikely that all these effects are downstream consequences of direct action of the C protein on the membranes. This can be achieved either by damaging the membrane integrity during the C protein oligomerization at the membrane surface or by formation of the pore-like structures. In line with this, the importance of the transmembrane domain and the immature form of the C protein for ER stress responses has been demonstrated in two separate studies. First, a mutational analysis with a truncated form corresponding to the mature C protein (1–177) revealed that a full-length core is required for induction of ER stress [57]. Second, expression of the C protein in mouse cells that are deficient in SPP and ubiquitin ligase TRC8, and hence unable to process the C protein into the mature form, was associated with increased ER stress [57].

## 5. Conclusions

**Figure 6:**
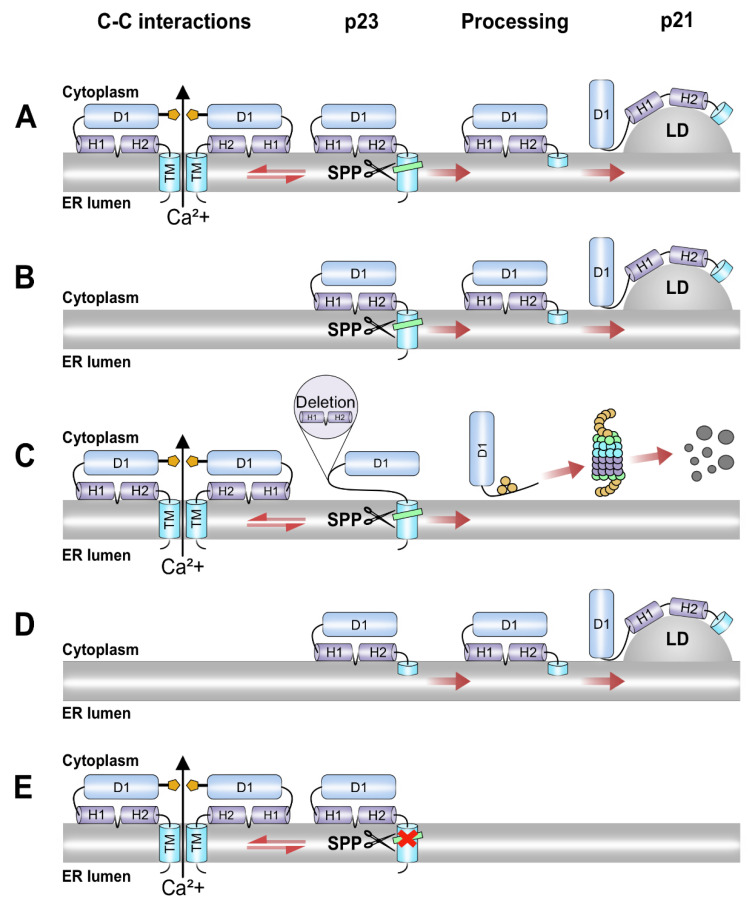
Model for activation of Ca^2+^ signaling by HCV C. (**A**) The immature C (p23) protein with intact transmembrane (TM) domain (also known as D3) and capacity to self-associate into oligomeric structures at the ER membrane, enables Ca^2+^ efflux from ER into the cytosol. After p23 is processed by SPP, the mature p21 protein is transported to the lipid droplets. (**B**) The immature p23 with intact TM domain, but defective in C-C interactions, does not perturb the ER membrane and hinders Ca^2+^ efflux (e.g., ∆2–18, ∆61–68). The localization of the mature form (p21) to lipid droplets is retained. (**C**) The immature form p23 with deletions in domain 2 (e.g., ∆124–142, ∆147–161) allows membrane perturbation prior to processing, but is rapidly degraded after maturation by SPP. (**D**) The C proteins with truncated TM domain (e.g., 1–173) do not allow Ca^2+^ efflux but are transported into lipid droplets. (**E**) The intermediate p23 deficient in SPP cleavage (e.g., spmt, ∆81–123) is retained in the ER membrane and is hyperactive for Ca^2+^ efflux.

Our present model of how Ca^2+^/NFAT signaling is triggered by the HCV C protein is summarized in Figure 6. In line with the proposed model, targeting of the HCV C protein to the ER is a prerequisite but not sufficient for inducing Ca^2+^/NFAT signaling, and is consistent with a model where proteolytic intermediates of the C protein with an intact transmembrane ER-anchor assemble into pore-like structures in the ER membrane. The biological function of this mechanism remains to be established, although it cannot be excluded that the physiological effects are side-effects of its replication process rather than being beneficial for the virus in evolutionary terms.

## Figures and Tables

**Table 1:** Construction of HCV core mutants.

Plasmid	Sequence of Inserted Oligonucleotide	Enzyme Sites Used for Insertion
pHCVC∆2–12	gaaGAATTCcaccATGcgtaacaccaaccgtcg	Eco RI ^a^, Avr II ^a^
pHCVC∆2–25	ctaaGAATTCcaccATGggcggtcagatcgttg	Eco RI ^a^, Avr II ^a^
pHCVC∆26–57	caaaCTCGAGgacccgggaacttgacg	Nde I ^b^, Xho I
pHCVC∆61–68	caaagCTCGAGgtcgtcggcccgagggcag	Eco RI ^a^, Avr II ^a^
pHCVC∆70–79	caaGGTACCCacgtgccttggggatagg	Nde I ^b^, Kpn I
pHCVC∆81–123	cagccgg/cgatacc	Kpn I, Cla I
pHCVC∆124–142	aggtcatcgaGCGGCCGCttggaggcgct	Cla I, Xag I
pHCVC∆147–161	cgtcggcgccCCTcttggAGGcgtgaactatgcaacagg	Nde I ^b^, Xag I
pHCVC1–161	ctaaGAATTCagccgtcttccagaaccc	Eco RI ^a^, Eco RI ^a^
pHCVC1–173	gaaGAATTCaagagcaaccaggaagg	Eco RI ^a^, Eco RI ^a^
pHCVC1–182	gaaGAATTCagagcagggccagaagg	Eco RI ^a^, Eco RI ^a^

^a^ Enzyme site located in the pCEN vector. ^b^ Enzyme site located in the pOP/CMV vector.

## Data Availability

Not applicable.
